# Supplementary material for: A terpene synthase-cytochrome P450 cluster in Dictyostelium discoideum produces a novel trisnorsesquiterpene
Source: eLife. 2019 May 7;8:e44352. doi: 10.7554/eLife.44352 (PMC6524965; doi:10.7554/eLife.44352)
Supplement: Supplementary file 1. [file elife-44352-supp1.docx]

**Supplementary file 1. NMR data of discoidol recorded in C6D6.**

| C^[a]^ | ^13^C (δ)^[b]^ | ^1^H (δ, m, *J*, int)^[c]^ |
| --- | --- | --- |
| 1 | 32.5 (CH_2_) | 2.27 (m, 1H)  1.98 (m, 1H) |
| 2 | 22.9 (CH_2_) | 1.52 (m, 1H)  1.47 (m, 1H) |
| 3 | 30.7 (CH_2_) | 1.93 (dddd, ^2^*J*=13.7, ^3^*J*=13.6, ^3^*J*=4.7, ^3^*J*=4.7, 1H)  1.24 (m, 1H) |
| 4 | 42.1 (CH) | 1.49 (m, 1H), |
| 5 | 39.1 (C_q_) | – |
| 6 | 39.9 (CH) | 1.68 (m, 1H),  1.18 (dd, ^3^*J*=13.6, ^3^*J*=13.6, 1H) |
| 7 | 43.7 (CH) | 1.67 (m, 1H), |
| 8 | 26.7 (CH_2_) | 1.99 (m, 1H)  1.67 (m, 1H) |
| 9 | 121.5 (CH) | 5.57 (ddd, ^3^*J*=6.8, ^3^*J*=1.8, ^4^*J*=1.8, 1H) |
| 10 | 141.0 (C_q_) | – |
| 11 | 71.8 (C_q_) | – |
| 12 | 27.2 (CH_3_) | 1.009 (s, 3H) |
| 13 | 27.4 (CH_3_) | 1.012 (s, 3H) |
| 14 | 30.6 (CH_3_) | 1.18 (s, 3H) |
| 15 | 17.9 (CH_3_) | 1.015 (d, ^3^*J*=6.9, 3H) |
| [a] Carbon numbering as shown in Figure 1. [b] Chemical shifts δ in ppm. [c] Chemical shifts δ in ppm, multiplicity m (s=singlet, d=doublet, m=multiplet), coupling constants *J* are given in Hertz. | | |

[α]_D_^20^ = +12.7 (*c* 0.32, (^2^H_6_)benzene). IR (diamond ATR): ν ̃ = 3384 (br, m), 2961 (s), 2928 (s), 2864 (s), 1667 (w), 1463 (s), 1373 (s), 1261 (m), 1219 (m), 1132 (w), 1092 (m), 1019 (s), 971 (m), 912 (m), 866 (s), 840 (m), 804 (w), 740 (s) cm^‑1^.
