# Supplementary material for: A terpene synthase-cytochrome P450 cluster in Dictyostelium discoideum produces a novel trisnorsesquiterpene
Source: eLife. 2019 May 7;8:e44352. doi: 10.7554/eLife.44352 (PMC6524965; doi:10.7554/eLife.44352)
Supplement: Supplementary file 2. [file elife-44352-supp2.docx]

**Supplementary file 2. Cytochrome p450 genes in *Dictyostelium discoideum*.**

| Gene name | Gene ID | Open reading frame |
| --- | --- | --- |
| *CYP524A1* | DDB_G0269016 | Full_length |
| *CYP513A3* | DDB_G0270354 | Full_length |
| *CYP516B1* | DDB_G0271778 | Full_length |
| *CYP519C1* | DDB_G0272556 | Full_length |
| *CYP515A1* | DDB_G0272704 | Full_length |
| *CYP525A1* | DDB_G0272652 | Full_length |
| *CYP508A1* | DDB_G0273943 | Full_length |
| *CYP508A3* | DDB_G0273047 | Full_length |
| *CYP508A2* | DDB_G0272604 | Full_length |
| *CYP519A1* | DDB_G0274523 | Full_length |
| *CYP518B1* | DDB_G0275197 | Full_length |
| *CYP518A1* | DDB_G0277545 | Full_length |
| *CYP513F1* | DDB_G0278679 | Full_length |
| *CYP513D1* | DDB_G0279763 | Full_length |
| *CYP51* | DDB_G0279403 | Full_length |
| *CYP513A1* | DDB_G0282183 | Full_length |
| *CYP515B1* | DDB_G0282283 | Full_length |
| *CYP513E1* | DDB_G0282353 | Full_length |
| *CYP522A1* | DDB_G0282769 | Full_length |
| *CYP508B1* | DDB_G0282419 | Full_length |
| *CYP517A1* | DDB_G0283929 | Full_length |
| *CYP517A4* | DDB_G0283933 | Full_length |
| *CYP519B1* | DDB_G0284089 | Full_length |
| *CYP556A1* | DDB_G0284345 | Full_length |
| *CYP508A4* | DDB_G0284535 | Full_length |
| *CYP517A2* | DDB_G0284647 | Full_length |
| *CYP554A1* | DDB_G0284923 | Full_length |
| *CYP519E1* | DDB_G0286419 | Full_length |
| *CYP555A1* | DDB_G0286743 | Full_length |
| *CYP513B1* | DDB_G0287087 | Full_length |
| *CYP519D1* | DDB_G0291448 | Full_length |
| *CYP520B1* | DDB_G0291702 | Full_length |
| *CYP516A1* | DDB_G0292168 | Full_length |
| *CYP520A1* | DDB_G0292496 | Full_length |
| *CYP508D1* | DDB_G0292790 | Full_length |
| *CYP508C1* | DDB_G0292792 | Full_length |
| *CYP521A1* | DDB_G0293738 | Full_length |
| *CYP514A2* | DDB_G0294561 | Full_length |
| *CYP514A4* | DDB_G0290707 | Full_length |
| *CYP514A1* | DDB_G0290743 | Full_length |
| *CYP513C1* | DDB_G0291105 | Full_length |
| *CYP508E1* | DDB_G0270904 | Full_length |
| *CYP519H1P* | DDB_G0279031 | pseudogene |
| *CYP513G1P* | DDB_G0279631 | pseudogene |
| *CYP513A2P* | DDB_G0290981 | pseudogene |
| *CYP513E2P* | DDB_G0282347 | pseudogene |
| *CYP513E3P* | DDB_G0282349 | pseudogene |
| *CYP508B2P* | DDB_G0293080 | pseudogene |
| *CYP518A2P* | DDB_G0273349 | pseudogene |
| *CYP519C2P* | DDB_G0280617 | pseudogene |
| *CYP515A2P* | DDB_G0272959 | pseudogene |
| *CYP517A3P* | DDB_G0271224 | pseudogene |
| *CYP516B2P* | NA | pseudogene |
| *CYP516A2P* | NA | pseudogene |
| *CYP514A3P* | NA | pseudogene |
