# Supplementary material for: A terpene synthase-cytochrome P450 cluster in Dictyostelium discoideum produces a novel trisnorsesquiterpene
Source: eLife. 2019 May 7;8:e44352. doi: 10.7554/eLife.44352 (PMC6524965; doi:10.7554/eLife.44352)
Supplement: Supplementary file 3. [file elife-44352-supp3.docx]

**Supplementary file 3. *CYP* genes of *Dictyostelium discoideum* that show significant coexpression with *DdTPS8*.**

| **Gene name** | **Pearson correlation coefficient** | **p** |
| --- | --- | --- |
| *CYP521A1* | .994** | 0 |
| *CYP508C1* | .992** | 0 |
| *CYP519C1* | .951** | 0.001 |
| *CYP516A1* | .941** | 0.002 |
| *CYP525A1* | .789* | 0.035 |
