# Supplementary material for: A terpene synthase-cytochrome P450 cluster in Dictyostelium discoideum produces a novel trisnorsesquiterpene
Source: eLife. 2019 May 7;8:e44352. doi: 10.7554/eLife.44352 (PMC6524965; doi:10.7554/eLife.44352)
Supplement: Supplementary file 4. [file elife-44352-supp4.docx]

**Supplementary file 4. Cytochrome p450 genes in *Dictyostelium purpureum*.**

|  |  |  |
| --- | --- | --- |
| Gene ID | Gene name | Note |
| DICPUDRAFT_93794 | *CYP51D1_Dp* | Full length |
| DICPUDRAFT_26606 | *CYP508A2_Dp* | Full length |
| DICPUDRAFT_156647 | *CYP508A4_Dp* | Full length |
| DICPUDRAFT_43840 | *CYP508A1_Dp* | Full length |
| DICPUDRAFT_146927 | *CYP508B1_Dp* | Full length |
| DICPUDRAFT_41061 | *CYP508B1_Dp* | Full length |
| DICPUDRAFT_92912 | *CYP508C1_Dp* | Full length |
| DICPUDRAFT_83304 | *CYP508D1_Dp* | Full length |
| DICPUDRAFT_77397 | *CYP508E1_Dp* | Full length |
| DICPUDRAFT_83390 | *CYP508A3_Dp* | Full length |
| DICPUDRAFT_54607 | *CYP508A1_Dp* | Full length |
| DICPUDRAFT_45676 | *CYP508B1_Dp* | Full length |
| DICPUDRAFT_97847 | *CYP508A2_Dp* | Full length |
| DICPUDRAFT_87778 | *CYP508A4_Dp* | Full length |
| DICPUDRAFT_25344 | *CYP513H1_Dp* | Full length |
| DICPUDRAFT_95724 | *CYP513H1 _Dp* | Full length |
| DICPUDRAFT_57144 | *CYP513A3_Dp* | Full length |
| DICPUDRAFT_74512 | *CYP513C1_Dp* | Full length |
| DICPUDRAFT_74514 | *CYP513C1_Dp* | Full length |
| DICPUDRAFT_150587 | *CYP513D1_Dp* | Full length |
| DICPUDRAFT_149158 | *CYP513F1_Dp* | Full length |
| DICPUDRAFT_80941 | *CYP513F1_Dp* | Full length |
| DICPUDRAFT_147217 | *CYP513A3_Dp* | Full length |
| DICPUDRAFT_74510 | *CYP513A3_Dp* | Full length |
| DICPUDRAFT_31424 | *CYP513D1_Dp* | Full length |
| DICPUDRAFT_42183 | *CYP514A1_Dp* | Full length |
| DICPUDRAFT_80939 | *CYP514A1_Dp* | Full length |
| DICPUDRAFT_80940 | *CYP514A4_Dp* | Full length |
| DICPUDRAFT_42091 | *CYP515A1_Dp* | Full length |
| DICPUDRAFT_99050 | *CYP515B1_Dp* | Full length |
| DICPUDRAFT_81297 | *CYP515B1_Dp* | Full length |
| DICPUDRAFT_153109 | *CYP516A1_Dp* | Full length |
| DICPUDRAFT_92019 | *CYP516B1_Dp* | Full length |
| DICPUDRAFT_37923 | *CYP518A1_Dp* | Full length |
| DICPUDRAFT_51478 | *CYP518A1_Dp* | Full length |
| DICPUDRAFT_96856 | *CYP519A1_Dp* | Full length |
| DICPUDRAFT_29455 | *CYP519B1_Dp* | Full length |
| DICPUDRAFT_29435 | *CYP519D1_Dp* | Full length |
| DICPUDRAFT_54169 | *CYP524A1_Dp* | Full length |
| DICPUDRAFT_54851 | *CYP519E1_Dp* | Full length |
| DICPUDRAFT_84371 | *CYP519H1P_Dp* | Full length |
| DICPUDRAFT_28572 | *CYP525A1_Dp* | Full length |
| DICPUDRAFT_17677 | *CYP555A1_Dp* | Full length |
| DICPUDRAFT_76441 | *CYP556A1_Dp* | Full length |
| DICPUDRAFT_80286 | *CYP519E1_Dp* | Full length |
| DICPUDRAFT_92221 | *CYP508B1_Dp* | Full length |
| DICPUDRAFT_154414 | *CYP508C1_Dp* | Full length |
| DICPUDRAFT_159750 | *CYP508B1_Dp* | partial |
| DICPUDRAFT_21969 | *CYP513H1_Dp* | partial |
| DICPUDRAFT_38755 | *CYP508A1_Dp* | partial |
| DICPUDRAFT_38792 | *CYP515A2_Dp* | partial |
| DICPUDRAFT_35628 | *CYP515A2_Dp* | partial |
| DICPUDRAFT_37832 | *CYP516B1_Dp* | partial |
| DICPUDRAFT_76460 | *CYP519E1_Dp* | partial |
