# Supplementary material for: A terpene synthase-cytochrome P450 cluster in Dictyostelium discoideum produces a novel trisnorsesquiterpene
Source: eLife. 2019 May 7;8:e44352. doi: 10.7554/eLife.44352 (PMC6524965; doi:10.7554/eLife.44352)
Supplement: Supplementary file 5. [file elife-44352-supp5.docx]

**Supplementary file 5. Primers used in gene cloning and vector construction**

|  |  |  |
| --- | --- | --- |
| **Gene name** | **Primer** | **Sequences** |
| *redB F* | Forward | 5’-CATATGGAAATTTTAGAATCAATTGATTTTATTG-3' |
| *redB R* | Reverse | 5'-CTCGAGTTAAAACCAAACATCTTGTAAATATCTAC-3' |
| *CYP521A1 F* | Forward | 5'-GGATCCGATGATTTTATTAACACTTTTATATTTAATTAT-3' |
| *CYP521A1 R* | Reverse | 5'-CTGCAGTTATCTTAATTTTAAATCAACTAAAAATG-3' |
| *CYP508C1 F* | Forward | 5'-GGATCCGATGGAATTATTAAATTCTTTACTGTTATTA-3' |
| *CYP508C1 R* | Reverse | 5'-CTGCAGTTATCTTGATTCTAAATTAATTTTATATTTA-3' |
| *BSTF2* | Forward | 5’-GTAGGGAGTTGATTTCAGACTATGCAC-3’ |
| *DdTPS8F* | Forward | 5’-ATGGATTATGATATAAAATTTACTTGGGA-3’ |
